# Candida keratitis and endopthalmitis after corneal transplantation; two case reports, a novel regimen and literature overview of therapeutic options

**DOI:** 10.1177/11206721211060140

**Published:** 2021-11-23

**Authors:** LC van der Wekken-Pas, PJ de Haas, R Wisse, M Rados, KCM van der Elst

**Affiliations:** 1 Department of Infectious Disease, University Medical Center Utrecht, Utrecht, the Netherlands; 2 Department of Microbiology, 8124 UMC Utrecht, the Netherlands; 3 Department of Ophthalmology, 8124 UMC Utrecht, the Netherlands; 4 Department of Clinical Pharmacy, Division of Laboratory Medicine and Pharmacy, 8124 UMC Utrecht, the Netherlands

**Keywords:** Keratitis, endophtalmitis, fungal infection, antifungals, pharmacotherapy, corneal transplantation

## Abstract

**Purpose:**

To evaluate treatment options for candida keratitis and endopthalmitis after corneal transplantation.

**Methods:**

Case reports and literature review.

**Results:**

Two patients with keratitis due to *Candida glabrata*/*parapsilosis* after corneal transplantation were successfully treated with a combination of topical voriconazole, intracameral voriconazole and amphotericin B, and systemic treatment with flucytosine.

**Conclusions:**

Natamycine and voriconazole topically are preferred therapeutic options for the treatment of fungal keratitis. Systemic flucytosine is a useful alternative additive, particularly for countries where natamycine is not registered as a pharmaceutical agent.

## Introduction

Fungal keratitis or endopthalmitis after corneal transplantation is a rare complication. Between 2007 and 2010 the Eye Bank Association of America reported 1.4 cases per 10,000 corneal transplants performed in that period.^1^ A single surgical centre analysed outcomes of the 99 Descemet stripping automated endothelial keratoplasties, performed on their patients between 2012 and 2014, and found 7 donor rim cultures which were positive for fungi. Two (28.6%) of 7 these patients developed interface fungal keratitis.^2^ The TRIP bio- and hemovigilance committee of the European Commission reported 7 fungal infections after usage of donor material, while 37542 tissues were used for transplantation.^3^

A review of 42 clustered cases shows that fungal post transplantation infections with *Candida* species are most often reported. The most common transmission mode was donor to host.^4^

There are no international guidelines to direct optimal therapy for fungal keratitis orendopthalmitis in general, let alone after corneal transplantation. We therefore felt these case reports and a review of the literature for these rare infections post corneal transplantation was needed. A statement of consent to publication of their images was gathered in both patients. This research received no specific grant from any funding agency in the public, commercial, or not-for-profit sectors.

## Case 1

A 73-year old woman underwent a routine corneal transplantation of the right eye because of Fuchs Endothelial Cell Dystrophy, specifically a posterior lamellar descemet membrane endothelial keratoplasty combined with phacoemulsification with insertion of an intraocular lens. The procedure was uneventful. Postoperatively she received prophylactic ofloxacin and dexamethasone eye drops and prednisolone ointment four times a day according to the local protocol. However, on the first postoperative day, cultures of the transport medium showed fungal growth. On the second postoperative day these cultures showed to be positive for *Candida glabrata (C. glabrata).* The patient reported no vision loss and slit lamp examination only showed corneal edema normal for the short-post surgical follow-up, without overt signs of infection or inflammation. Prophylactic antifungal treatment with voriconazole eye drops (10 mg/mL, administrated every hour) and oral voriconazole (two times daily 400 mg on the first day, followed by two times daily 200 mg) was started. Because of interaction between oral voriconazole and statin and cardiac antiarrhythmic medication used by the patient, and because the cultures of the transport medium showed a minimal inhibitory concentration (MIC) of 0.12 μg/mL for voriconazole, oral voriconazole was stopped on the sixth postoperative day. Treatment with ocular voriconazole was continued and tapered to eight times daily.

**Figure 1.**
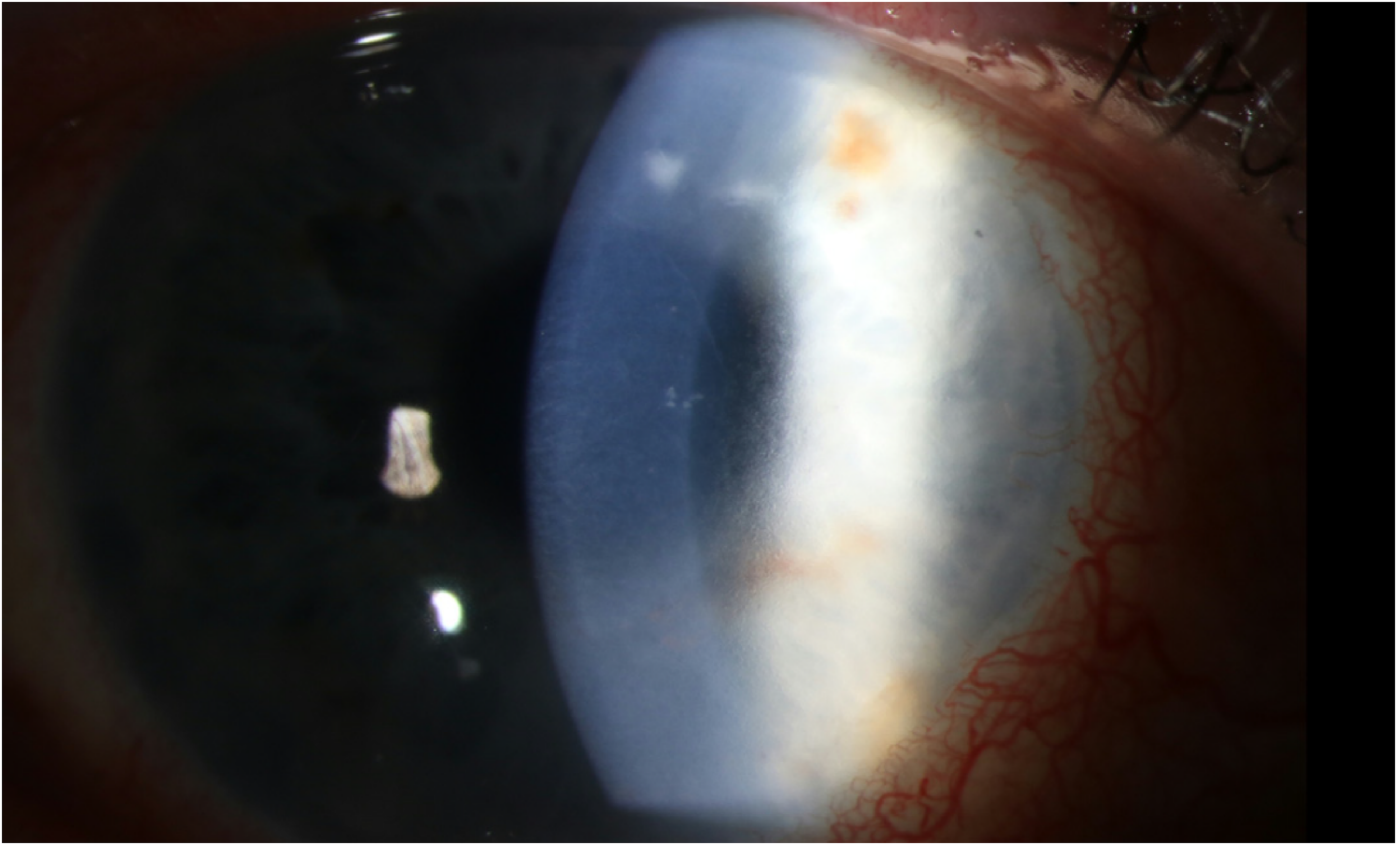
Case 1. Slit lamp examination, 17 days after corneal transplantation, during ocular voriconazole treatment.

**Figure 2.**
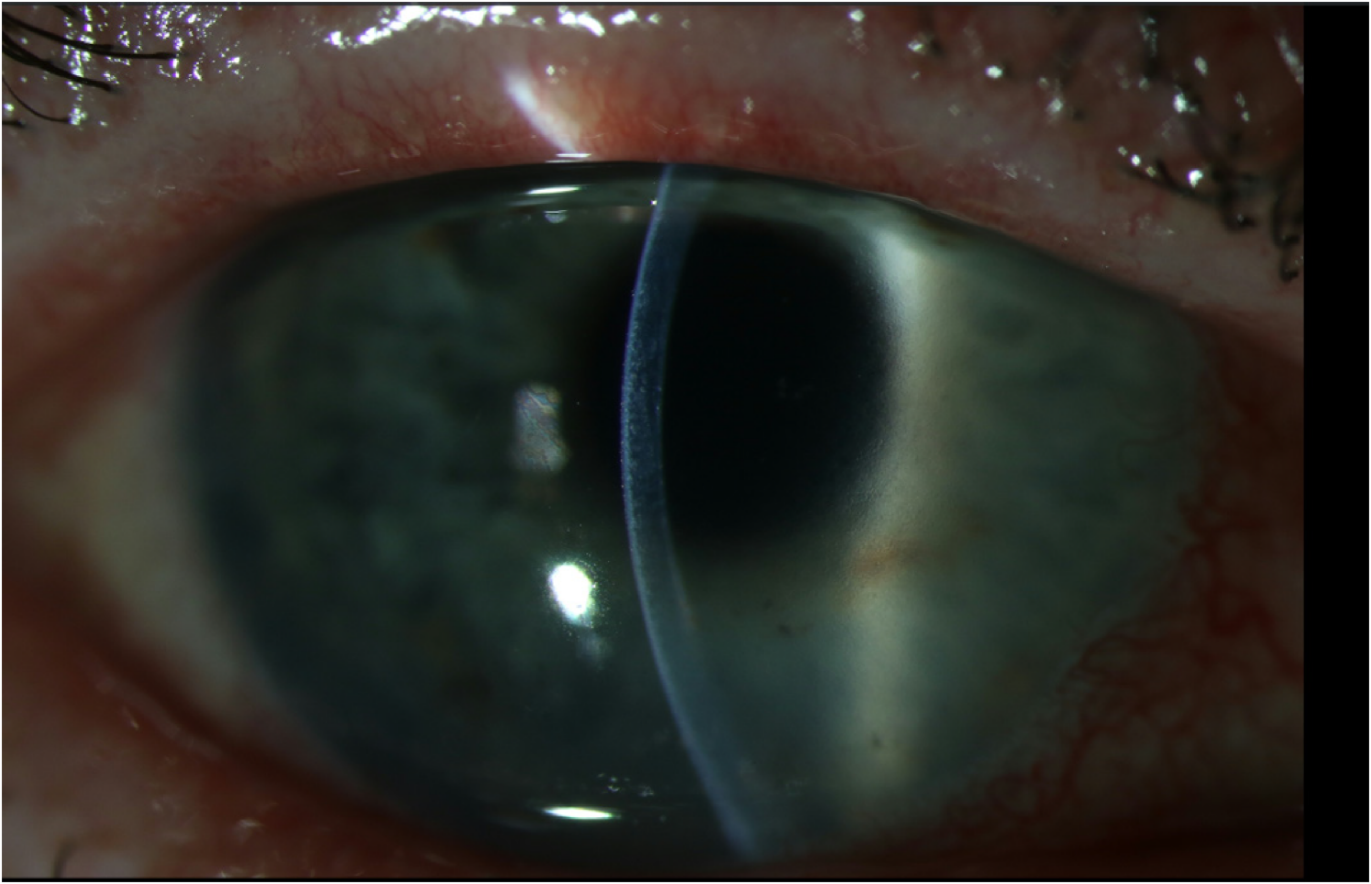
Case 1. Slit lamp examination, 17 days after corneal transplantation, during ocular voriconazole treatment.

On the 20^th^ postoperative day, the patient developed visual complaints of the right eye. Slit lamp examination showed a small velvety infiltrate, epitheliopathy, corneal edema and 2 + cells in the anterior eye chamber with a positive Tyndall effect (see Figures 1 and 2). Cultures of anterior eye chamber fluid confirmed the diagnosis of keratitis with *C. glabrata* despite topical and earlier systemic voriconazole treatment.

On the 21st day after the operation, amphotericin B (5 μg/0.1 mL) and voriconazole (100 μg/0.1 mL) were injected intracamerally. This was repeated initially every 2 days. After the sixth intracameral injection this was slowly tapered, three further injections were given at an interval of 4 days and a final tenth injection was given five days after. At the same time, on the 22nd postoperative day, systemic treatment with intravenous flucytosine was added to the treatment regimen in a dose of 25 mg/kg four times daily (and switched to oral treatment on the 40^th^ postoperative day and after adequate intake and blood levels were assured). Blood levels of flucytosine were obtained weekly and were measured using a validated liquid chromatography-mass spectrometry method. The plasma trough concentration was therapeutic and varied between 33.8 and 53.1 mg/L, which were deemed adequate.^5^ The flucytosine level in fluid obtained from the anterior eye chamber was 60.9 mg/L while the plasma trough concentration on the same day was 53.1 mg/L, which suggests an excellent penetration of the drug in the fluid of the anterior eye chamber. No local or systemic side effects were noted.

Three weeks after the first intracameral injection and after initiation of flucytosine, slit lamp examination showed a clear transplant, no cells in the anterior eye chamber and two densities in the deep stroma, less prominent then during the previous examination, suspect for scar tissue. Repeated cultures of the intravitreal fluid were negative. Intracameral injections were stopped and voriconazole eye drops were reduced from 8 to 4 times daily. Due to further improvement one month later, flucytosine was stopped as well. Two weeks later, no further signs of the infection were noted and the voriconazole eye drops were discontinued as well. The corneal graft remained clear after three months of follow-up and the patients best corrected visual acuity was 0.9, with no manifest signs of endothelial cell failure.

## Case 2

**Figure 3.**
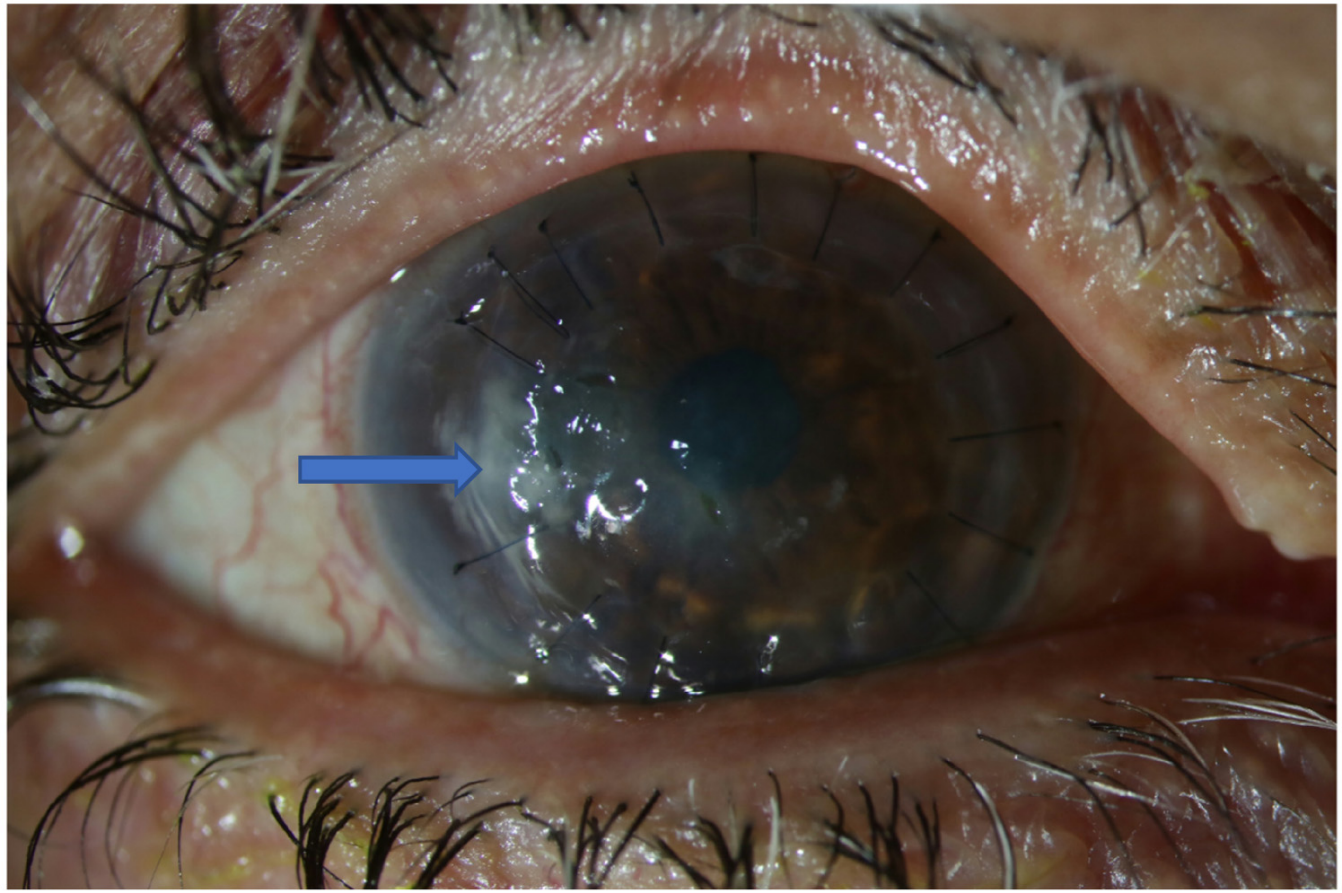
Case 2. During treatment with topical voriconazole. Arrows denoting infiltrates.

**Figure 4.**
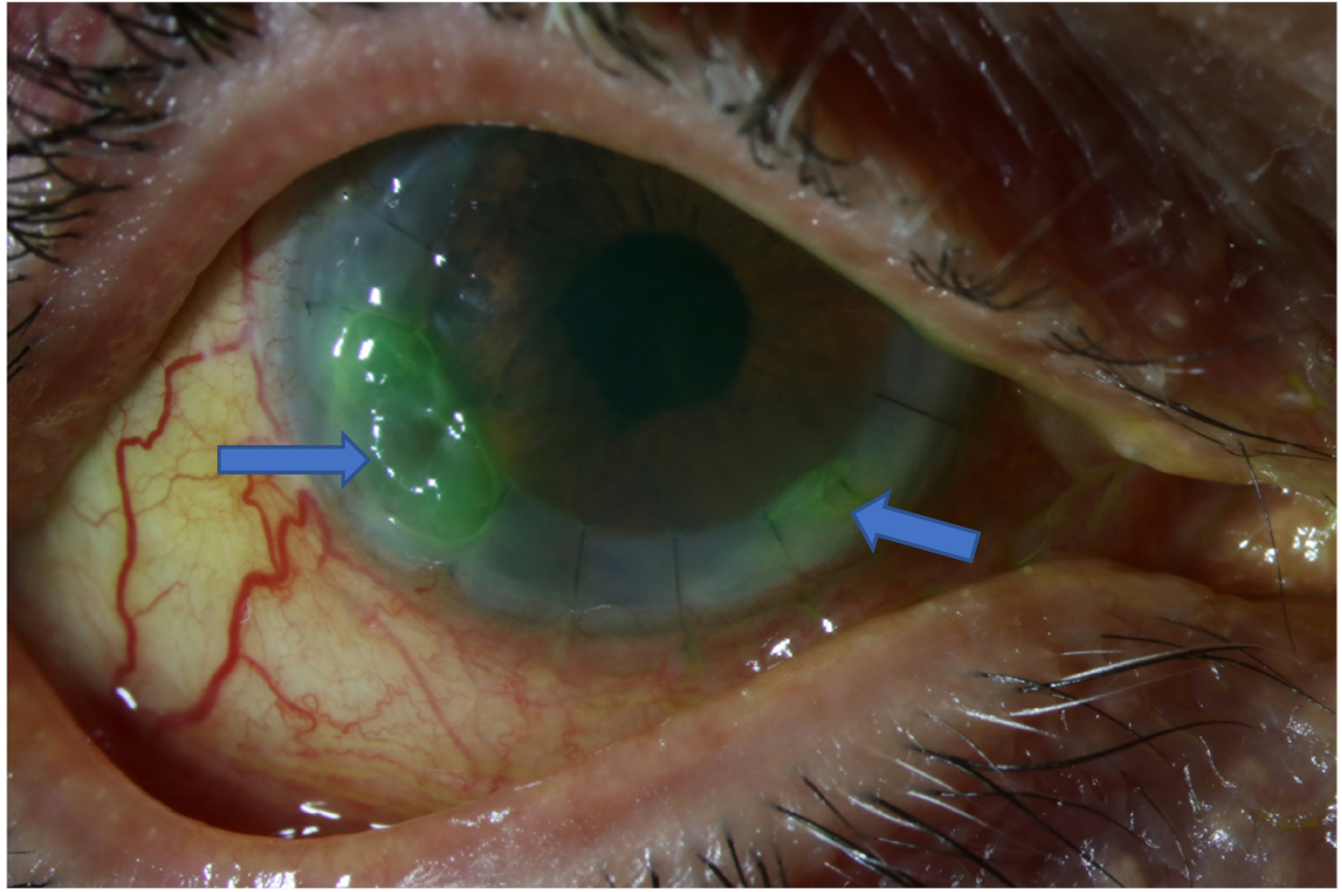
Case 2. Two months after regraft. Arrows denoting infiltrates.

A 61-year old patient with diabetes mellitus type 1, and a hereditary anterior corneal dystrophy, underwent a corneal transplantation of the right eye, specifically a deep anterior lamellar keratoplasty. Six months after transplantation the patient complained of blurry vision and discharge from the right eye. No trauma to the eye was reported. A central corneal erosion was identified with slit lamp examination together with multiple neighbouring small granulomatous corneal lesions. Corneal swab cultures were positive with *Candida parapsilosis.* Initial treatment with chlorhexidine 8 times daily showed insufficient improvement. Voriconazole 1% eyedrops 8 times daily was started, but because no response was noted after 2.5 months a perforating keratoplasty was scheduled to attack the infection (Figures 3 and 4). Voriconazole was continued postoperatively, however, after one month of follow up visual acuity did not improve and edema of the transplant was noted, with a small erosion and no cell reaction in the anterior chamber.

Cultures of fluid of the anterior eye chamber were positive with *C. parapsilosis* (MIC voriconazole <0.008 μg/mL), where after intracameral injections with amphotericin B (10 μg/0.1 mL) and voriconazole (100 μg/0.1 mL) were given and systemic flucytosine (25 mg/kg, four times daily) was started. Plasma trough concentration of flucytosine varied between 16 and 47.7 mg/L. The concentration in the fluid of the anterior eye chamber was 42.9 mg/L with a plasma trough concentration of 47.7 mg/L.

After six intracameral injections with amphotericin B and voriconazole, and five weeks of systemic flucytosine in combination with topical voriconazole, the erosion had diminished and repeated cultures of anterior eye chamber fluid were negative, after which only topical voriconazole was continued for two more months with good clinical results. Visual acuity continued to be low however, without improvement after YAG (yttrium-aluminium-garnet) capsulotomy.

Two months after discontinuation of the antimycotic treatment, the patient continued to have blurred vision, intra-ocular pressure slowly increased and eventually an endopthalmitis with *C. parapsillosis* was diagnosed. A vitrectomy was performed and voriconazole 200 mg two times daily and flucytosine 25 mg/kg were administered systemically, amphotericin B 5μg/0.1 mL was additionally given twice intravitreously. After six weeks, the patient showed clinical improvement and flucytosine was discontinued. Voriconazole was continued one month longer.

One month after discontinuation of voriconazole, the patient developed pain, photophobia, and a red right eye. Anterior chamber fluid cultures did not show any growth*,* voriconazole was however restarted prophylactically both systemically and topically as was natamycine topically. Under this medication, a new corneal infiltrate developed. With the suspicion of a bacterial superinfection moxiflocaxin 5 mg/mL eyedrops 8 times a day was started. The corneal swab showed growth of a *Staphylococcus aureus*. Good clinical improvement was seen after one month. Voriconazole was tapered and moxifloxacine was discontinued. However, two months later, again a severe inflammation of the right eye developed. Anterior chamber fluid cultures were again positive for *C. parapsillosis,* susceptible for voriconazole*.* Voriconazole systemically was restarted, and amphotericin B was again given intravitreously.

Currently, the patient is still being treated with voriconazole systemically, with an adequate therapeutic response, and a continuous chronic systemic treatment with voriconazole is considered.

## Discussion

Whether an antifungal agent is suitable to treat fungal keratitis and endopthalmitis depends mainly on its ability to penetrate the affected parts of the eye. The unique anatomical properties of the eye and the biochemical characteristics of the antifungal drug create a therapeutic challenge.

### Voriconazole

Voriconazole belongs to the family of azoles and interferes in the ergosterol synthesis which is essential for cell membrane integrity of the fungal cell. Several studies show good penetration of topical applied voriconazole eye drops 10 mg/mL, with drug concentrations in the fluid of the anterior eye chamber ranging from 3.3–6.5 mg/L.^6,7^ For this reason, voriconazole was continued in our patient despite the MIC of 0.12.

The use of systemic voriconazole has been tested in the MUTT (mycotic ulcer trial) 2 trial, but showed no additional benefit, although side effects were more frequent (vision disturbances and liver biochemical changes).^8^

Some case series report good clinical outcome after stromal injection of 1.0% voriconazole in combination with other systemic and topical antifungal agents,^9,10^ although no benefit on top of topical natamycine was found in two randomised clinical trial in patients with keratitis predominantly caused by *Fusarium* and *Aspergillus spp*. Unfortunately, no patients with keratitis caused by *Candida* species were included.^11,12^

### Amphotericin B

The use of amphotericin B is not widely studied in fungal keratitis. Systemic administration of this polyene does not lead to therapeutic concentrations in the eye.^13^ Some anecdotal evidence exists for topical amphotericin B, but due to methodological issues and the small populations studied, it is not advisable to use it in the treatment of fungal keratitis.^14,15^

Intracameral and intrastromal injection of amphothericin B in recalcitrant fungal keratitis (no resolution or aggravation after one week of 0.5% fluconazole every half hour, combined with 5% natamycin or 0.25% amphotericin B every two hours, and 200 mg of oral itraconazole daily) was beneficial in a small study in nine patients with keratitis caused by *Fusarium spp*, *Aspergillus spp* and *Alternaria spp*..^16^ Comparable results were found in three cases with corneal ulcer and hypopyon due to *Aspergillus* despite treatment with topical natamycin 5%, amphotericin B 0.15%, and oral itraconazole, after they had undergone intracameral injections with 10 μg in 0.1 mL amphotericin B.^17^

Natamycin is another polyene, predominantly used and studied in Asia. Natamycin is not registered in the European Union for use in human, but is widely used in veterinary care and food preservation.

A systematic review of eight randomised controlled trials with a total of 793 patients showed better visual acuity after treatment with topical natamycine in comparison with topical voriconazole (weighted mean difference 0.13, 95% confidence interval (CI) 0.00–0.27) and fewer patients required a therapeutic keratoplasty after treatment with natamycin (RR (relative risk) 1.89, 95% CI: 1.14–3.12).^18^ These results were confirmed in another systematic review.^19^

Intracameral injections with amphotericin B leads to comparable results as topical natamycin,^12^ but the possible complications of such injections and known toxicity^20^ makes natamycin a more preferable option.

### Echinocandins

Echinocandins are known to have very low biological availability in the central nervous system and eye-tissues after systemic administration. Topical use was studied in rabbits with *C. albicans* keratitis and showed microbiological response (decrease in CFU) and decrease in ulcus surface with the use of caspofungin 0.15%.^21^ A clinical study in 29 patients with candida keratitis showed comparable results in the group treated with topical micafungin 0.1% and fluconazole 0.2%.^22^

### Flucytosine

A final treatment option might be flucytosine. Topical administration of this drug does not result in therapeutic concentrations in the other tissue layers of the eye. Although this might change when there is inflammation due to infection.

Pharmacokinetic studies with systemic flucytosine have been performed in rabbits, showing adequate flucytosine concentrations in the fluid of the anterior and posterior eye chamber after systemic administration.^23,24^ Clinical evidence consist of a few case reports.^25^ Our cases demonstrate good clinical and microbiological response after intracameral treatment in combination with systemic flucytosine. The adequate flucytosine concentration in the fluid of the anterior eye chamber underscores the potential of this drug in the treatment of fungal keratitis, although monotherapy with flucytosine should be avoided, because of the risk of emerging resistance.

## Conclusions

Fungal keratitis and endophtalmitis via contaminated culture medium is a very rare complication of a corneal transplantation. Treatment options are diverse, though high level of evidence for any treatment option is lacking. Locally administered natamycine and voriconazole seem clinically the most effective therapy for keratitis and have favourable toxicity profiles, but natamycine is unavailable for treatment in Europe.

The presented cases show that matching levels of flucytosine in the anterior chamber and serum are obtainable with systemic flucytosine treatment. With careful dosing the toxicity of flucytosine is limited, and this drug therefore may be a potential additive in the treatment for fungal keratitis and endopthalmitis.
